# A risk prediction model for evaluating thrombosis extension of muscle calf venous thrombosis after craniotomy

**DOI:** 10.3389/fsurg.2022.992576

**Published:** 2022-10-14

**Authors:** Juhua Li, Huayu Chen, Mei Liu, Zheng Lin, Xingzhen Ren, Ying Wang, Xingchen Zou, Zejuan Gu

**Affiliations:** ^1^ Department of Neurosurgery, The First Affiliated Hospital of Nanjing Medical University, Nanjing, China; ^2^ Department of Neurosurgery, Children's Hospital of Nanjing Medical University, Nanjing, China

**Keywords:** craniotomy, thrombosis extension, muscle calf venous thrombosis, prediction model, nursing

## Abstract

**Objective:**

To explore the risk factors of muscle calf venous thrombosis (MCVT) after craniotomy and construct a risk prediction model, so as to provide tool for evaluating the prognosis of MCVT after craniotomy.

**Methods:**

Retrospective analysis was performed on the data of patients undergoing craniotomy complicated with MCVT from January 1, 2018 to December 31, 2020. A prediction model was established by Logistic regression, and the predictive efficacy of the model was tested by ROC curve. The accuracy of the risk model was evaluated by Hosmer-Lemeshow (H-L) test, and the model was verified internally by cross validation.

**Results:**

Among the 446 patients who underwent craniotomy complicated with MCVT, 112 cases (25.11%) had thrombosis extension. D-dimer, Capirini scores, length of hospital stay, malignant tumor, fracture, use of dehydrating agents and hemostatic agents were independently related to thrombosis extension after craniotomy. The area under ROC curve (AUROC) of the prediction model was 0.918 (0.888, 0.942), and the sensitivity and specificity of the maximum Youden index were 85.3% and 78.2%, respectively. H-L test showed that the prediction model was accurate (*χ*^2^ = 12.426, *P* = 0.133). The internal verification results of the prediction model showed that the AUROC value of the prediction model is 0.892.

**Conclusion:**

The prediction model has a good prediction efficacy on the prognosis of post-craniotomy patients complicated with MCVT, and can be used as a tool to evaluate the risk of thrombosis extension.

## Introduction

The incidence of deep venous thromboembolism (DVT) in patients after craniotomy in neurosurgery is as high as 16%–34% (1), which can be divided into central type, peripheral type, and mixed type according to the formation site. Muscle calf venous thrombosis (MCVT), which is a peripheral DVT, refers to a thrombus originating in the soleus muscle or gastrocnemius vein. The most common clinical manifestations of MCVT are local tenderness of the leg, without obvious swelling, pain, and other conscious symptoms (2). Studies have shown that the calf intermuscular vein is not only the prone site of DVT but also the most common embolic origin of pulmonary thromboembolism (3). Porfidia et al. found that without intervention, the probability of MCVT spreading to peripheral veins and proximal veins was as high as 16.3%–30.4% (4–6). As for MCVT, relevant studies at home and abroad mainly focus on the influencing factors and nursing of postoperative MCVT formation, and there are few reports on the establishment and application of the risk prediction model of thrombosis spread.

In this study, the risk factors for the spread of new MCVT after craniotomy were explored, independent clinical predictors were identified and a prediction model for the spread of MCVT was established in order to predict the risk of the spread of MCVT in clinical practice.

## Methods

### Patient recruitment

A total of 446 patients with newly diagnosed MCVT after craniotomy from Jiangsu Province Hospital from January 1, 2018 to December 31, 2020 were recruited. All patients received homogenous perioperative preventive DVT education. Inclusion criteria: (1) 18–80 years old; (2) Patients initially diagnosed with MCVT after craniotomy. Exclusion criteria: (1) Patients with pre-existing thrombosis and/or diagnosed pulmonary embolism before admission. (2) Patients with coagulation disorder. (3) Patients with incomplete information, patients who gave up treatment or died after enrollment. This study has been approved by the Ethics Committee of Jiangsu Province Hospital, and all the enrolled patients have signed informed consent. Considering the simplicity and applicability of the model, the model included at most 10 risk factors. According to the modeling sample size calculation formula requirements (7), it is assumed that each factor needs 10 cases for verification. According to literature review, the spread rate of MCVT is about 25%. Considering 10% sample loss, therefore, the sample size required for this study is 10 × 10 × (1 + 0.1) ÷ 0.25 = 440 cases.

### Diagnostic criteria

Color Doppler ultrasound has high accuracy and noninvasive diagnosis of lower extremity deep vein thrombosis, and is the preferred method for DVT diagnosis (8). In this study, MCVT was all examined and confirmed by the attending physicians in the department of ultrasound. The typical sonogram (9) showed that the venous lumen could not be completely compressed after the probe was pressed, and the local area was obviously dilated and pearly or rope-like low echo was found inside. New MCVT patients were reviewed by ultrasound every 3 days to determine the development of thrombosis. If spread occurs, relevant treatment will be carried out until the discharge of guidance.

### Data collection

All patient data were collected by the study group members through the hospital's electronic medical record management system and examination system. We consult paper medical records when necessary. Double-check and input software to ensure complete and accurate information. Data collected on patients included: (1) Basic information: gender, age, height, weight, body mass index, length of stay; (2) Clinical data: disease diagnosis, complications, GCS score, name of the operation, duration of operation, muscle strength, anticoagulant therapy, application of dehydrators, application of hemostatic drugs, bedtime, time and location of thrombosis; (3) Laboratory tests: biochemical indicators, coagulation indicators on the first day after surgery, D-dimer, blood routine, etc.

### Statistical analysis

SPSS22.0 software was used for statistical analysis. The normal distribution of quantitative data was described by mean ± standard deviation, and *t*-test was used to compare groups. Non-normal distributions were described by median (interquartile spacing) and rank-sum tests were used to compare groups. Qualitative data were expressed as frequency and percentage (%), and *χ*^2^ test was used for comparison between groups. The risk prediction model was constructed by multi-factor analysis using binary Logistic regression. The area under ROC curve was used to predict the performance, and the test level *α *= 0.05. Hosmer-lemeshow goodness of fit test was used to evaluate the calibration degree of the prediction model. If the test result showed *P* > 0.05, there was a small difference between the predicted value of the model and the observed value, suggesting that the prediction model had a good calibration degree and high accuracy of the model.

## Results

**Figure 1:**
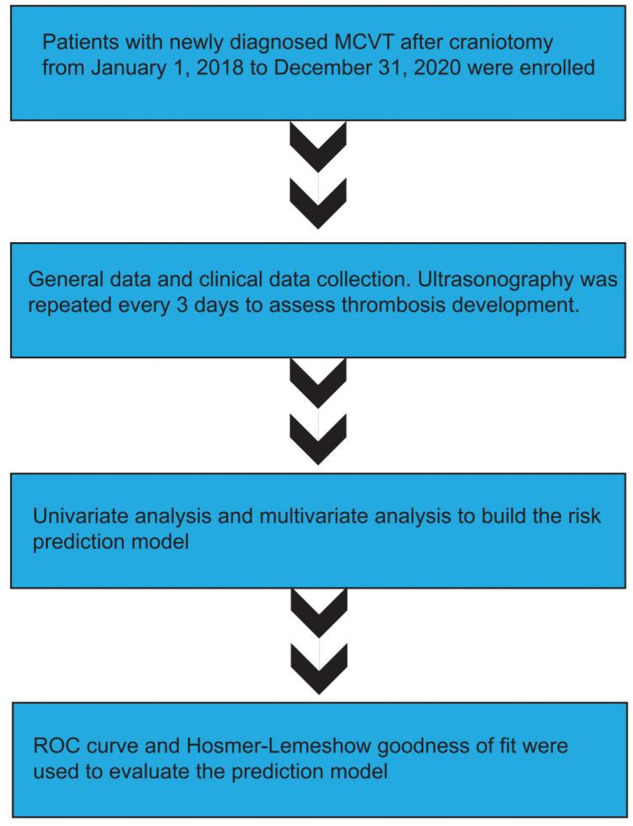
The flow chart of our study.

The workflow of our study was shown in Figure 1.

### Basic information of the patient

A total of 446 patients with new MCVT after craniotomy, aged 62.12 ± 0.53 years, were included in this study, including 237 males (53.2%) and 209 females (46.8%). There were 234 cases of brain tumor, 106 cases of cerebrovascular disease, 67 cases of brain trauma and 39 cases of hydrocephalus. MCVT spread occurred in 112 patients (25.11%).

### Univariate analysis of actors influencing the spread of new MCVT after craniotomy

As shown in Table 1, age, BMI, platelet, alanine aminotransferase, alkaline phosphatase, prothrombin time (PT), internationalized standardized ratio of PT (PT-INR), D-dimer, Caprini score, affected side muscle strength, Glasgow Coma Scale GCS), length of hospital stay, history of alcohol consumption, history of smoking, complications (fracture, pulmonary infection, malignant tumor, dyskinesia), use of dehydrants, anticoagulant therapy, and use of hemostatic agents were associated with the spread of new MCVT thrombosis after craniotomy (*P* < 0.05).

**Table 1:** Univariate analysis.

Variable	No thrombus spread (*n* = 334)	Thrombus spread (*n* = 112)	*t*, *z*, *χ*^2^ values	*P*-value
Male (case)	172	64	1.02	0.313^a^
Age (years)	62.0 ± 11.2	65.3 ± 7.9	2.982	**<0.001** ^b^
BMI, kg/m^2^	24.42 ± 2.37	25.06 ± 1.85	2.608	0.003
White blood cells (×109/L)	8.56 ± 0.20	8.61 ± 0.39	−0.121	0.452^b^
Red blood cells (×1,012/L)	3.52 (3.10–3.89)	3.53 (3.17–3.97)	0.681	0.50^c^
Hemoglobin (g/L)	107.34 ± 18.13	103.78 ± 23.53	−1.660	0.098^b^
Platelet (×109/L)	202.50 (156.00–269.00)	181.00 (128.00–252.50)	2.565	**0.010** ^c^
ALT (U/L)	18.40 (13.30–31.45)	15.35 (11.00–26.45)	2.501	**0.012** ^c^
AST (U/L)	21.45 (17.60–30.10)	21.25 (15.85–28.75)	1.405	0.160^c^
Alkaline phosphatase (U/L)	84.35 (70.05–103.20)	76.10 (63.15–95.20)	2.722	**0.007** ^c^
L-γ glutamyl transpeptidase (U/L)	28.00 (17.50–47.90)	23.00 (16.95–40.90)	1.057	0.291^c^
Total bilirubin (mmol/L)	11.75 (8.70–16.60)	11.65 (8.75–14.80)	0.065	0.948^c^
Direct bilirubin (mmol/L)	4.30 (3.20–6.15)	4.35 (3.10–6.10)	0.091	0.927^c^
Indirect bilirubin (mmol/L)	7.40 (5.40–10.45)	7.10 (5.50–9.65)	0.327	0.744^c^
Total cholesterol (mmol/L)	4.43 ± 1.55	4.24 ± 1.16	−1.166	0.244^b^
Triglycerides (mmol/L)	1.29 (0.97–1.74)	1.13 (0.79–1.86)	1.238	0.216^c^
High-density lipoprotein (mmol/L)	1.01 (0.82–1.26)	1.07 (0.85–1.28)	0.913	0.361^c^
Low density lipoprotein (mmol/L)	2.59 (2.10–3.24)	2.65 (2.03–3.04)	0.513	0.608^c^
Albumin (g/L)	37.07 ± 4.97	36.71 ± 5.22	−0.631	0.528^b^
Prothrombin time (s)	13.08 ± 1.84	13.77 ± 3.14	2.827	**0.005** ^c^
PT-internationalization normalized ratio (PT-INR)	1.12 (1.05–1.20)	1.13 (1.06–1.26)	1.882	0.060^c^
Activated partial thromboplastin times (s)	29.10 (26.80–31.40)	29.50 (26.95–33.45)	1.702	0.089^c^
Thrombin time (s)	17.27 ± 4.67	16.99 ± 1.54	−0.622	0.534^b^
D-dimer	3.25 (1.38–8.15)	7.76 (3.67–13.56)	5.768	**<0.001** ^c^
Capirini socre	5 (4–7)	8 (7–9)	11.809	**<0.001** ^c^
hemiplegia	111, 33.2%	50, 44.6%	5.706	0.127^a^
Disease side muscle strength			9.730	**0.008** ^a^
Muscle strength level 5	152, 45.5%	41, 36.6%		
Muscle strength level 2–4	119, 35.9%	34, 30.4%		
Muscle strength level 0–1	63, 19.2%	37, 33.0%		
GCS socre	13 (10–15)	11 (8–13)	4.587	**<0.001** ^c^
Length of stay (days)	22 (16–29)	20 (14–24)	2.280	**0.023** ^c^
Stay in bed >7 days	52, 15.6%	16, 14.3%	0.031	0.861^a^
Drinking history	29	20	6.311	**0.012** ^a^
Smoking history	25	26	18.967	**<0.001** ^a^
Diabetes	46	19	0.454	0.501^a^
Hypertension	149	47	0.143	0.705^a^
Fracture	3	31	4.297	0.038^a^
Lung infection	125	76	30.159	**<0.001** ^a^
Tumor	8	24	42.810	**<0.001** ^a^
Movement disorders	91	53	14.558	**<0.001** ^a^
Dehydrant drugs			8.256	**0.041** ^a^
No use	129	28		
One type	179	72		
Two types	23	9		
Three types	3	3		
Use of hormone	243	98	9.329	0.143^a^
Anticoagulation	14	0		**0.025** ^a^
Use of hemostatic agents	131	69	16.100	**<0.001** ^a^

Continuous variables were expressed as “mean ± standard deviation” or “median (interquartile spacing)”.

^a^ Chi-square test or Fisher's exact probability method.

^b^ Independent sample *T*-test.

^c^ Mann–Whitney rank sum test.

Bold values represents statistical significance.

### Multivariate analysis of factors influencing the spread of new MCVT after craniotomy

Binary Logistic regression analysis was performed with the occurrence of MCVT spread (no = 0, yes = 1) as the dependent variable, and the statistically significant risk factors in univariate analysis as independent variables. The results showed that alkaline phosphatase, D-dimer, Capirini score, length of hospital stay, complication of fracture, complication of malignancy, and use of dehydrating agents and hemostatic agents were independent risk factors for the spread of MCVT (Table 2).

**Table 2:** Multivariate analysis.

Variable	*Β*-value	Standard error	OR	95% CI	*P*-value
Age	0.032	0.019	1.032	0.995–1.071	0.092
BMI	0.1261	0.08392	1.1344	0.962–1.337	0.133
Platelet (×109/L)	−0.001983	0.001677	0.9980	0.994–1.001	0.237
ALT	0.003666	0.006005	1.0037	0.991–1.015	0.541
ALP	−0.01467	0.006602	0.9854	0.972–0.998	**0.026**
PT	0.06196	0.03939	1.0639	0.984–1.149	0.115
PT-INR	0.3149	0.8468	1.3701	0.260–7.203	0.710
D-dimer	0.07197	0.02147	1.0746	1.030–1.120	**<0.001**
Capirin score	0.7515	0.1053	2.1203	1.724–2.606	**<0.001**
Muscle strength	0.05011	0.3062	1.0514	0.577–1.915	0.870
GCS score	0.04125	0.06179	1.0421	0.923–1.176	0.504
Length of stay (days)	−0.05786	0.01899	0.9438	0.909–0.979	**0.002**
Drinking history	−0.1357	0.8303	0.8731	0.171–4.444	0.870
Smoking history	0.6684	0.7272	1.9510	0.469–8.114	0.358
Fracture	−3.7088	1.3025	0.0245	0.001–0.314	**0.004**
Lung infection	0.1518	0.4069	1.1639	0.524–2.583	0.709
Tumor	2.9515	0.6921	19.1340	4.927–74.293	**<0.001**
Movement disorders	0.4155	0.4119	1.5151	0.675–3.396	0.313
Dehydrant drugs	−0.865	0.306	0.421	0.231–0.766	**0.004**
Use of hemostatic agents	0.9612	0.3926	2.6147	1.211–5.645	**0.014**

Bold values represents statistical significance.

### Construction and internal validation of a prediction model for the spread of new MCVT after craniotomy

Based on the results of univariate analysis analysis and multivariate analysis, the prediction model was constructed through logistic regression. The model results were shown in Table 3. The risk prediction model formula was as follows:

Logit(P)=−5.742+0.067X1+0.75X2−0.047X3−0.742X4+1.183X5+2.731X6−2.136X7.

**Table 3:** Logistic regression model.

Variable	*Β*-value	OR	95% CI	*P*-value
Constant	−5.742	0.003	0.001–0.012	**<0.001**
D-dimer	0.067	1.069	1.030–1.110	**<0.001**
Capirini score	0.750	2.117	1.795–2.497	**<0.001**
Length of stay	−0.047	0.954	0.924–0.985	**0.004**
Dehydrant drugs	−0.742	0.476	0.286–0.793	**0.004**
Use of hemostatic agents	1.183	3.264	1.651–6.455	**0.001**
Tumor	2.731	15.350	5.024–46.897	**<0.001**
Fracture	−2.136	0.118	0.022–0.641	**0.013**

Bold values represents statistical significance.

Risk of MCVT spread in individuals:

$$P=\frac{e^{-5.742+0.067X1+0.75X2-0.047X3-0.742X4+1.183X5+2.731X6-2.136X7}}{1+e^{-5.742+0.067X1+0.75X2-0.047X3-0.742X4+1.183X5+2.731X6-2.136X7}}.$$

**Figure 2:**
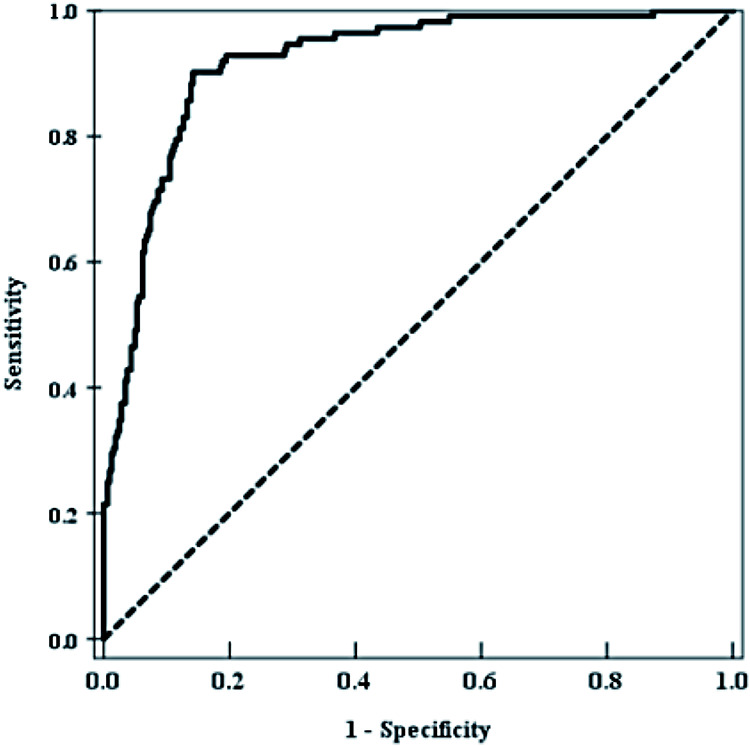
The distinguishing ability of models was evaluated by ROC curve.

The distinguishing ability of models was evaluated by ROC curve, as shown in Figure 2. In this study, the area under ROC curve was 0.918, 95% CI was 0.888–0.942, and the sensitivity and specificity at the maximum value of Youden index were 85.3% and 78.2%, respectively. H-L test showed that the calibration degree of the prediction model was high (*χ*^2^ = 12.426, *P* = 0.133), and the AUC value of the prediction model was 0.892. The results of subgroup analysis were shown in Supplementary Figure S1 and Supplementary Table S1.

## Discussion

The results of this study showed that age was positively correlated with the occurrence of MCVT thrombosis spread after craniotomy. In elderly patients, muscle atrophy, decreased venous vascular elasticity, decreased venous flap function, and lower limb blood flow velocity are slowed, so age is an important factor affecting the progression of venous thrombosis (10). This may be due to the fact that with the increase of age, the inner wall of blood vessels becomes more damaged and rough, and thrombosis is more prone to spread (11, 12). Therefore, it is of clinical significance to pay attention to new MCVT in elderly patients and carry out the early intervention.

Patients with higher BMI have a higher risk of MCVT spread, which may be caused by abnormal metabolism in obese patients, resulting in abnormal endogenous coagulation and fibrinolytic system, and hypercoagulability (13). This study found that the longer prothrombin time, the higher the risk of MCVT spread. Some studies have found that abnormal coagulation indicators are correlated with DVT formation, which may be related to the activation of the endogenous coagulation system by abnormal coagulation (14–16). D-dimer is the main indicator for laboratory examination of acute VTE at present. The quantitative ELISA method is mostly used for detection, and its level is not only affected by DVT but also affected by the pregnancy, surgery, infection, tumor, trauma, etc., resulting in high sensitivity and low specificity (17). The increase of D-dimer can enhance the effect of the fibrinolytic enzyme, make fibrin form specific degradation products, and increase the chance of DVT formation by 3.5 times (18). This study found that the increase of D-dimer was positively correlated with the risk of MCVT spread. Studies have reported that up to 35% of MCVT patients may have normal D-dimer levels, suggesting that the diagnostic sensitivity and specificity of this test to exclude MCVT is lower than that of proximal DVT (19). Some studies have also suggested that a higher D-dimer level indicates a higher possibility of complications and a sudden increase in the risk of early death (20).

Caprini thrombosis risk assessment model is a scale for risk assessment of thrombosis with good effectiveness (21, 22). In this study, the score of the MCVT spread group was higher than that of the non-spread group, indicating that the score of the Caprini scale is meaningful for the risk prediction of MCVT spread after craniotomy. In addition, MCVT patients with paraplegia, especially those with strength below grade 3, were at higher risk of thrombosis spread. Consistent with previous research results, the reason may be that decreased muscle strength leads to prolonged bed rest, muscle weakness or even atrophy of lower limbs, and failure to complete normal muscle pump activities, resulting in slow venous blood flow and blood stasis, high blood coagulation, and exacerbating thrombosis and progression. GCS score is used to assess the severity of patients' disturbance of consciousness (23). The data analysis in this group found that the lower the GCS score is, the higher the risk of MCVT spread. The reason may be that the more serious nerve function injury is, the more likely it is to be accompanied by a limb movement disorder and promote blood stasis and thrombotic progression (24).

Our results showed that smoking history and drinking history were influential factors for the spread of new MCVT after craniotomy, and patients with smoking history and drinking history were more likely to have thrombosis spread. Considering that patients with a history of smoking are more likely to develop lung infection, a large number of inflammatory mediators are released, promoting platelet activation and exacerbating blood hypercoagulability, neurosurgical craniotomy patients are at high risk for the spread of new MCVT (25, 26). Studies have also shown that the relationship between alcohol intake and increased or decreased risk of VTE is largely undetermined (27). Therefore, more studies are needed to confirm the relationship between drinking history and the incidence of new MCVT spread after craniotomy.

According to the results of this study, patients with fractures or dyskinesia are at increased risk of MCVT spread. This may be due to the slow blood flow of lower limbs after fracture or movement disorder, resulting in platelet aggregation at the injured site and promoting the formation and spread of thrombosis.

And for the patients with pulmonary infection or tumors with high MCVT spread risk, it may be due to the increased expression of inflammatory factors, activating the coagulation system in the body and leading to an imbalance in the body's blood coagulation/anticoagulant system (28, 29). It can also inhibit the fibrinolytic system and prevent the degradation of fibrin, thus inducing the spread of thrombosis (29). Therefore, for patients with fractures or dyskinesia, the braking time should be shortened as soon as possible, and bedside rehabilitation exercises should be increased. For patients with pulmonary infection or malignant tumor, active and effective anti-infection or anti-tumor treatment can effectively reduce the risk of thrombosis and spread.

The choice of treatment is also an important factor influencing the prognosis of MCVT. Dehydrating agents and diuretics are needed in the acute stage of cerebral edema in patients with craniotomy, which leads to blood concentration and promotes thrombosis and progression. Multivariate analysis showed that the use of dehydrating agent was an independent risk factor for the spread of MCVT. Therefore, the early prevention of thrombosis and progression in patients who use a large number of dehydrators is significant. The results of this study indicate that anticoagulant therapy is an influential factor in the spread of MCVT, and patients with new MCVT without anticoagulant therapy are more likely to have the spread. However, anticoagulant therapy for MCVT is currently controversial in clinical practice (30, 31). Some scholars suggested that anticoagulant therapy can prevent the progression of thrombosis and reduce the incidence of pulmonary embolism. Some scholars also believe that anticoagulant therapy after craniotomy has a 2%–4% risk of intracranial hemorrhage and MCVT can dissolve itself, so anticoagulant therapy is not advocated. It has been suggested (32) that intravenous ultrasound monitoring may be an alternative to systematic anticoagulant therapy for patients at low risk of thrombotic spread.

In this study, both univariate analysis and Logistic regression analysis showed that the use of hemostatic agents was a risk factor for the spread of new MCVT after craniotomy. The reason may be that the use of hemostatic drugs can make blood vessel contraction, activate the coagulation system, promote platelet aggregation, and accelerate the adverse progress of thrombosis. It is suggested that in clinical work, medical staff not only need to assess the risk of postoperative bleeding in patients with craniotomy but also need to assess whether patients have high-risk factors for the spread of MCVT, to more effectively screen high-risk patients and provide a basis for early intervention.

Studies have shown that 70%–90% of emboli of pulmonary embolism come from lower limb veins with thrombosis (33), among which MCVT accounts for up to 15%–25%. Some studies (4) found that 20.3% of isolated MCVT patients had thrombosis spread within 3 months, and 6.5% of them spread to the popliteal vein level, which was similar to the results of this study. After craniotomy, the risk of thrombosis spread is greatly increased due to the long operation time, the application of dehydrating agent, consciousness disorder, decreased ability of limb movement, longer bedtime, and other reasons, which not only affects the disease outcome of craniotomy patients but also increases the risk of death. In this group, only 1 patient developed pulmonary thromboembolism during hospitalization, which was not life-threatening. 19 cases of MCVT spread to the popliteal vein level, timely placement of filter treatment. Previous studies on MCVT were mostly limited to the exploration of risk factors, and there are few studies on the spread of MCVT. The establishment of the risk prediction model for new MCVT spread after craniotomy is helpful for medical staff to pay attention to the prevention of MCVT spread. Through this model, we can intuitively display the relationship between risk factors and postoperative MCVT spread in the formula. It is helpful for medical staff to pay attention to the potential risk of MCVT spread in patients and provide corresponding intervention measures for existing risk factors of MCVT spread in patients, such as timely correction of thrombocytopenia, and liver function protection and timely intervention for patients with decreased muscle strength. Therefore, the establishment of a prediction model with clinical applicability is of great significance for the early identification and prevention of the spread of MCVT. However, our study also has some limitations. We studied only individual independent risk factors for MCVT and did not systematically develop a formula to calculate overall risk. Secondly, we lack corresponding prospective studies and randomized clinical trials to confirm our results, and we will improve it in the future.

## Conclusion

The risk prediction model of MCVT spread after craniotomy constructed in this study has good predictive efficacy, and can provide a risk assessment tool for the prognosis of MCVT complicated after craniotomy. However, there are limitations to our study. This study was a single-center study with limited sample representativeness. Compared with the prospective study, this study is a retrospective study with a certain bias, and it may be biased to select only venous ultrasound results as the basis for diagnosis of MCVT. Multi-center and large-sample studies are expected to be carried out in the future to further improve the model and provide a reference for early clinical identification of high-risk groups for the spread of MCVT.

## Data Availability

The original contributions presented in the study are included in the article/**Supplementary Material**, further inquiries can be directed to the corresponding author/s.
