# Supplementary material for: A risk prediction model for evaluating thrombosis extension of muscle calf venous thrombosis after craniotomy
Source: Front Surg. 2022 Oct 14;9:992576. doi: 10.3389/fsurg.2022.992576 (PMC9614109; doi:10.3389/fsurg.2022.992576)
Supplement: Supplementary file 1 [file Table1.docx]

Table 1. Univariate analysis

| **Variable** | **No thrombus spread**  **(n = 334)** | **Thrombus spread**  **(n=112)** | ***t、z、ꭓ^2^  values*** | ***P-value*** |
| --- | --- | --- | --- | --- |
| Male (case) | 172 | 64 | 1.02 | 0.313^a^ |
| Age (years) | 62.0±11.2 | 65.3±7.9 | 2.982 | **<0.001 ^b^** |
| BMI，kg/m^2^ | 24.42±2.37 | 25.06±1.85 | 2.608 | 0.003 |
| White blood cells (x 10^9^ / L) | 8.56±0.20 | 8.61±0.39 | -0.121 | 0.452 ^b^ |
| Red blood cells (x 10^12^ / L) | 3.52(3.10-3.89) | 3.53(3.17-3.97) | 0.681 | 0.50^c^ |
| Hemoglobin (g/L) | 107.34±18.13 | 103.78±23.53 | -1.660 | 0.098 ^b^ |
| Platelet (×10^9^/L) | 202.50(156.00-269.00) | 181.00  (128.00-252.50) | 2.565 | **0.010 ^c^** |
| ALT(U/L) | 18.40(13.30-31.45) | 15.35(11.00-26.45) | 2.501 | **0.012 ^c^** |
| AST(U/L) | 21.45(17.60-30.10) | 21.25(15.85-28.75) | 1.405 | 0.160 ^c^ |
| Alkaline phosphatase(U/L) | 84.35(70.05-103.20) | 76.10(63.15-95.20) | 2.722 | **0.007 ^c^** |
| L-γ glutamyl transpeptidase(U/L) | 28.00(17.50-47.90) | 23.00(16.95-40.90) | 1.057 | 0.291 ^c^ |
| Total bilirubin(mmol/L) | 11.75(8.70-16.60) | 11.65(8.75-14.80) | 0.065 | 0.948 ^c^ |
| Direct bilirubin(mmol/L) | 4.30(3.20-6.15) | 4.35(3.10-6.10) | 0.091 | 0.927 ^c^ |
| Indirect bilirubin(mmol/L) | 7.40(5.40-10.45) | 7.10(5.50-9.65) | 0.327 | 0.744 ^c^ |
| Total cholesterol(mmol/L) | 4.43±1.55 | 4.24±1.16 | -1.166 | 0.244^b^ |
| Triglycerides(mmol/L) | 1.29(0.97-1.74) | 1.13(0.79-1.86) | 1.238 | 0.216 ^c^ |
| High-density lipoprotein(mmol/L) | 1.01(0.82-1.26) | 1.07(0.85-1.28) | 0.913 | 0.361 ^c^ |
| Low density lipoprotein(mmol/L) | 2.59(2.10-3.24) | 2.65(2.03-3.04) | 0.513 | 0.608 ^c^ |
| Albumin(g/L) | 37.07±4.97 | 36.71±5.22 | -0.631 | 0.528^b^ |
| Prothrombin time(s) | 13.08±1.84 | 13.77±3.14 | 2.827 | **0.005 ^c^** |
| PT-internationalization normalized ratio (PT-INR) | 1.12(1.05-1.20) | 1.13(1.06-1.26) | 1.882 | 0.060 ^c^ |
| Activated partial thromboplastin times(s) | 29.10(26.80-31.40) | 29.50(26.95-33.45) | 1.702 | 0.089 ^c^ |
| Thrombin time(s_ | 17.27±4.67 | 16.99±1.54 | -0.622 | 0.534^b^ |
| D - dimer | 3.25(1.38-8.15) | 7.76(3.67-13.56) | 5.768 | **<0.001^c^** |
| Capirini socre | 5 (4-7) | 8 (7-9) | 11.809 | **<0.001 ^c^** |
| hemiplegia | 111, 33.2% | 50, 44.6% | 5.706 | 0.127 ^a^ |
| Disease side muscle strength |  |  | 9.730 | **0.008 ^a^** |
| Muscle strength level 5 | 152, 45.5% | 41, 36.6% |  |  |
| Muscle strength level 2-4 | 119, 35.9% | 34, 30.4% |  |  |
| Muscle strength level 0-1 | 63, 19.2% | 37, 33.0% |  |  |
| GCS socre | 13(10-15) | 11(8-13) | 4.587 | **<0.001 ^c^** |
| Length of stay (days) | 22(16-29) | 20(14-24) | 2.280 | **0.023 ^c^** |
| Stay in bed >7 days | 52, 15.6% | 16, 14.3% | 0.031 | 0.861 ^a^ |
| Drinking history | 29 | 20 | 6.311 | **0.012 ^a^** |
| Smoking history | 25 | 26 | 18.967 | **<0.001 ^a^** |
| Diabetes | 46 | 19 | 0.454 | 0.501 ^a^ |
| Hypertension | 149 | 47 | 0.143 | 0.705 ^a^ |
| Fracture | 3 | 31 | 4.297 | 0.038 ^a^ |
| Lung infection | 125 | 76 | 30.159 | **<0.001 ^a^** |
| Tumor | 8 | 24 | 42.810 | **<0.001^a^** |
| Movement disorders | 91 | 53 | 14.558 | **<0.001^a^** |
| Dehydrant drugs |  |  | 8.256 | **0.041 ^a^** |
| No use | 129 | 28 |  |  |
| One type | 179 | 72 |  |  |
| Two types | 23 | 9 |  |  |
| Three types | 3 | 3 |  |  |
| Use of hormone | 243 | 98 | 9.329 | 0.143 ^a^ |
| Anticoagulation | 14 | 0 |  | **0.025 ^a^** |
| Use of hemostatic agents | 131 | 69 | 16.100 | **<0.001 ^a^** |

Note: Continuous variables were expressed as "mean ± standard deviation" or "median (interquartile spacing)". A: Chi-square test or Fisher's exact probability method, B: Independent sample T-test, c: Mann-Whitney rank sum test
