# Supplementary figures and images for: A risk prediction model for evaluating thrombosis extension of muscle calf venous thrombosis after craniotomy
Source: Front Surg. 2022 Oct 14;9:992576. doi: 10.3389/fsurg.2022.992576 (PMC9614109; doi:10.3389/fsurg.2022.992576)

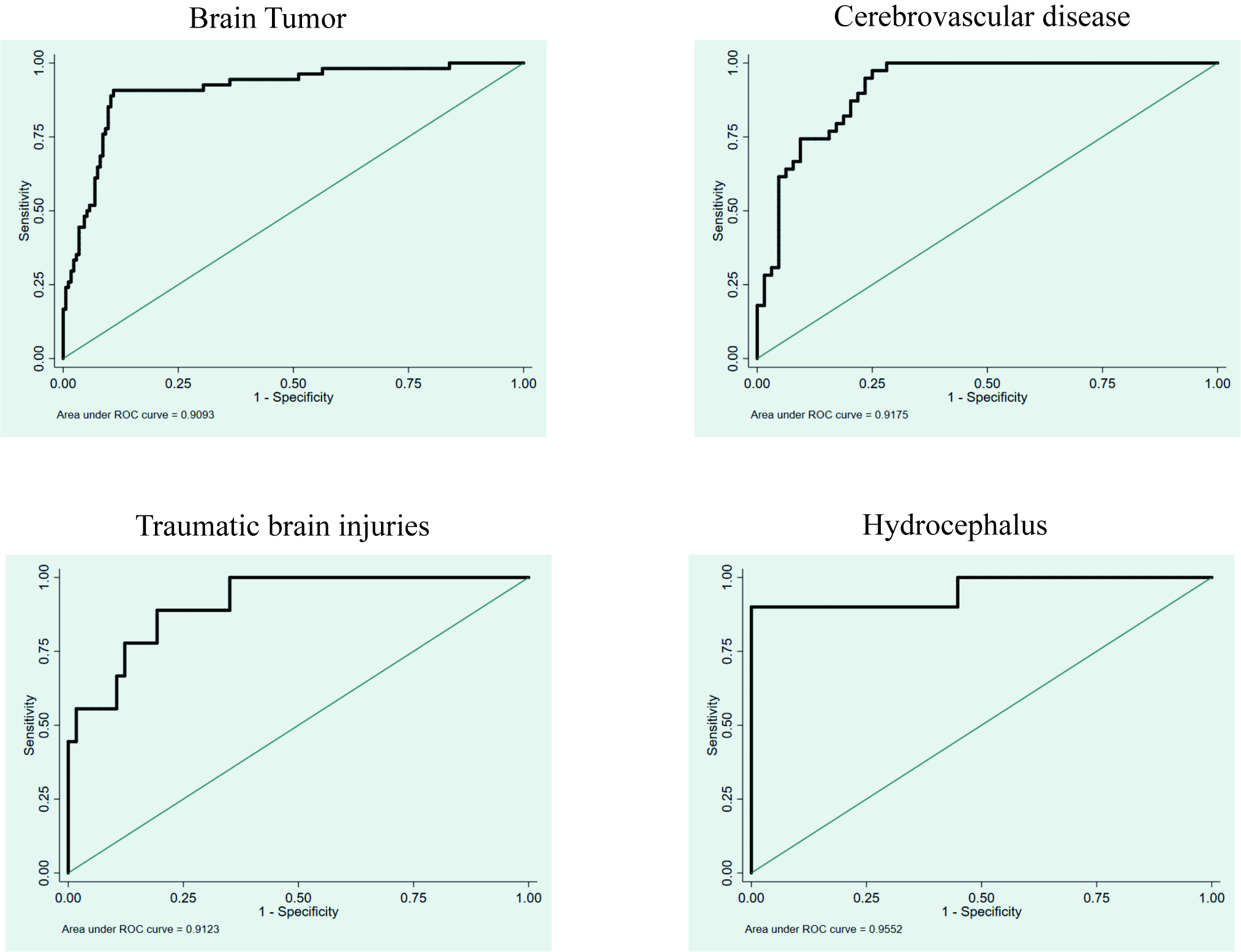

Supplement: Supplementary file 2 [file Image1.tif]
